# Influence of participation in a quality improvement collaborative on staff perceptions of organizational sustainability

**DOI:** 10.1186/s12913-020-06026-3

**Published:** 2021-01-07

**Authors:** James H. Ford, Aaron Gilson

**Affiliations:** University of Wisconsin – Madison School of Pharmacy, 777 Highland Ave, Madison, WI 53705 USA

**Keywords:** Sustainability, Quality improvement collaborative, Participation, Staff perceptions, NIATx, Intervention

## Abstract

**Background:**

Sustainability capacity (SC), which is an organization’s ability to implement and maintain change, is influenced by internal attributes, environmental contextual influencers, and intervention attributes. Temporal changes in staff SC perceptions, as well as the influence of quality improvement collaborative (QIC) participation, has generally not been explored. This project addresses this gap, measuring staff SC perceptions at four time points (baseline and every 9 months) for clinics participating in an intervention – the Network for the Improvement of Addiction Treatment QIC initiative (called NIATx200).

**Methods:**

A mixed linear model repeated measures analysis was applied to matched staff members (*n* = 908, representing 2329 total cases) across the evaluation timeframe. Three separate statistical models assessed potential predictors of SC perceptions: Time (Models I-III); NIATx200 intervention, staff job function, and tenure (Models II &III); and NIATx200 participation hours and four organizational variables (Model III).

**Results:**

For Model I, staff perceptions of total SC increased throughout most of the study (t_1,4_ = − 6.74, *p* < .0001; t_2,4_ = − 3.100, *p* < .036; t_3,4_ = − 0.23, p = ns). Model II did not change Model I’s overall Time effect, but combined NIATx200 services (t = − 2.23, *p* = .026), staff job function (t = − 3.27, *p* = .001), and organizational administrators (t = − 3.50, p = .001) were also significantly associated with greater perceptions of total SC. Inclusion of additional variables in Model III demonstrated the importance of a higher participation level (t = − 3.09, *p* < .002) and being in a free-standing clinic (t = − 2.06, *p* < .04) on staff perceptions of total SC.

**Conclusion:**

Although staff exposure to sustainability principals was minimal in NIATx200, staff perceptions about their organization’s SC significantly differed over time. However, an organization’s participation level in a QIC became the principal predictor of staff SC perceptions, regardless of other factors’ influence. Given these findings, it is possible to develop and introduce specific sustainability content within the structure of a QIC to assess the impact on staff SC perceptions over time and the sustainment of organizational change.

**Trial registration:**

ClinicalTrials.gov, NCT00934141. Registered July 6, 2009. Retrospectively registered.

**Supplementary Information:**

The online version contains supplementary material available at 10.1186/s12913-020-06026-3.

## Background

An organization’s sustainability capacity (SC) represents its ability to implement and maintain the benefits of a systems change over time [1]. Substance use treatment clinics provide services to individuals with an opioid use disorder or alcohol use disorder, including clinical counseling and access to medications, and many of these individuals have co-occurring mental health disorders [2, 3]. These clinics face unique challenges when trying to implement and sustain changes, such as disengaged staff, lack of organizational capacity to sustain change, unease with making changes, or administrative process barriers such as multiple phone calls to schedule an appointment [4–7]. It is important, therefore, to understand how substance use clinic staff perceive the likelihood that changes within their organization will be sustained.

Sustainability frameworks suggest that an organizations’ capacity to sustain change is influenced by multilevel factors or constructs related to organizational attributes, environmental contextual features, and intervention characteristics [1, 8–10]. Examples of organizational attributes include leadership support, champion roles, revised policies and procedures or expert coaching support; external contextual features relate to regulatory or financial changes; and innovation attributes focus on ease of use or understanding how likely the benefits of the change would be sustained. Multiple studies have shown that various organizational, external, and innovation attributes inform the likelihood of sustaining an evidence-based practice within an organization [11–19]. Efforts to sustain change within an organization are not only a function of its SC but also depends on staff involvement. Although leadership support and the role of a champion are factors or constructs within these frameworks, less emphasis is placed on the role of staff involvement in implementing and sustaining change. As such, little is known about how participation in change efforts influence staff perceptions of an organization’s SC changes over time.

### Sustainability capacity

Recent efforts sought to define and refine the classification of sustainability factors or constructs. The Integrated Sustainability Framework (ISF) identified 36 factors across multiple settings (e.g., community, school, clinical/social services) as being associated with sustainability [20]. These factors were grouped into five contexts: (a) outer context, (b) inner context, (c) intervention characteristics, (d) processes, and (e) implementer and population characteristics. Example ISF factors within each context include sociopolitical context and funding environment (outer context); funding/resources, staffing and turnover (inner context); adaptability, fit with population or context and benefits/need (intervention); partnership/engagement and program evaluation (process); and implementer motivation and attitudes (implementer/population) [20].

Alternatively, the Consolidated Framework for Sustainability Constructs (CFSC) conceptualized 40 constructs across six themes associated with sustainability of change in healthcare settings [21]. Themes include: (a) initiative design and delivery, (b) negotiations related to the initiative processes, (c) organizational setting, (d) people or individuals involved, (e) resources, and (f) external environment. The CFSC also explored approaches [retrospective (after the implementation has occurred) versus prospective (explored throughout implementation)] for assessing staff perceptions about sustainability and the level of focus [organizational (e.g., substance use provider) versus intervention (e.g., a single improvement project)] associated with the assessment of sustainability capacity [21]. The efforts resulted in the identification of the ten most prevalent sustainability constructs within each category (Additional File 1) [21]. Four constructs are common across both level of focus and assessment timing. These include: demonstrating effectiveness and monitoring progress over time (initiative design and delivery), leadership and champions (people involved), and general resources to support sustainability (resources) [21]. Other CFSC constructs varied according to whether they should be assessed at the organizational or intervention level. For example, training and capacity building, and integration with existing programs and policies were not typically assessed within an organizational level of focus; staff perceptions about the belief in the initiative is not assessed for the intervention level of focus and stakeholder participation in the retrospective approach [21].

### Staff perceptions of sustainability capacity

Conceptually, the ISF and CFSC frameworks treat sustainability capacity as a process by which certain organizational attributes such as leadership support or staff involvement influence how change is sustained in an organization. Despite extensive research on sustainability constructs and associated frameworks, few instruments have been developed and extensively utilized in research to quantitatively assess staff perceptions about organizational sustainability capacity associated with the constructs in the ISF or CFSC [22–24]. These constructs represent elements often included in the structure of a quality improvement collaborative (QIC), such as understanding how implementation support and improvement methods might interact with stakeholder participation in training and capacity building activities to influence staff perceptions about an organization’s ability to sustain change. Although the Normalization Process Theory established a framework for evaluating staff perceptions and participation [25], research has not, to date, explored staff perceptions about SC as an outcome measure and how participation in a QIC influences those changes. Further, sustainability instruments have not been utilized to prospectively assess how staff perceptions about sustainability change over time while participating in a QIC.

Our objective in this manuscript was to explore temporal changes in staff SC perceptions for individuals working in substance use providers, as measured by the British National Health Services Sustainability Index (BNHS-SI), and how participation in a Network for the Improvement of Addiction Treatment (NIATx) quality improvement collaborative (QIC), called NIATx200, influenced those changes. Data collected during the NIATx200 initiative was utilized to begin addressing this important implementation research issue. This analysis builds on a prior analysis [26] and seeks to answer the research question, “What staff and organizational characteristics predict sustainability levels across the study timeframe?” guided study design and sample selection. The specific aims of the current paper were to: (1) explore temporal changes in staff perceptions about sustainability and (2) assess how staff and organizational characteristics as well as organizational participation in their assigned implementation strategy within a QIC influence changes in sustainability over time.

## Methods

### Study setting: NIATx200

The NIATx200 initiative built on prior successful NIATx research [4, 27–30]. NIATx200 evaluated the effectiveness of implementation strategies commonly used a QIC. To achieve this objective, NIATx200 recruited 201 addiction treatment clinics in five states (Massachusetts, Michigan, New York, Oregon, and Washington). Clinic eligibility criteria included: 60+ admissions per year, outpatient or intensive outpatient levels of care as defined by the American Society of Addiction Medicine (ASAM); and received some public funding in the past year [31]. Clinics, randomized within states, were stratified by size (number of patients per year) and management score [32] and assigned to one of four implementation strategies: (1) interest circle calls (*n* = 49), (2) learning sessions (*n* = 54), (3) coaching (*n* = 50), or (4) a combination of all three implementation strategies (*n* = 48). The NIATx200 initiative consisted an 18-month active implementation timeframe. During three distinct implementation periods lasting 6 months, participating clinics implemented organizational changes designed to improve wait time (mean days between first contact and first treatment), retention in treatment (percent of patients retained from first to fourth treatment session), and annual admissions. Data was also collected at the staff level about their perceptions associated with organizational readiness for change and sustainability propensity. The structure of the NIATx200 initiative and the description of the implementation strategies are described in more detail elsewhere [31, 33, 34].

Mixed-effect regression models determined which implementation strategy was most effective in improving outcomes, as well as being most cost-effective [31]. Improvements in the wait time and admission outcomes for clinics assigned to the coaching and combination strategies significantly differed from clinics assigned to the interest circle strategy and the coaching strategy was the more cost-effective as compared to interest circles [34]. Although no NIATx implementation strategy significantly improved treatment retention (as defined), an exploratory analysis, accounted for early treatment drop-off (i.e., a client not making it to the first treatment session) when measuring retention, showed clinic-level improvements for providers assigned to the coaching, combination and learning session implementation strategies which suggest that how retention was defined impacted the findings [34]. Results from this exploratory analysis clearly indicated that clinic participation in the three intervention (i.e., learning sessions, coaching, and the combination arm) improved the outcomes, with coaching being the most cost-effective strategy.

Although differences in clinic attributes did not affect improvements in the outcomes examined in other studies, organizational characteristics were included in these secondary data analyses. Organizational characteristics comprised: (1) non-profit status, (2) whether the clinic was free-standing Alcohol and Drug Abuse Treatment Program or part of a healthcare system, (3) whether the clinic had received accreditation from a national organization such as the Joint Commission on Accreditation of Healthcare Organizations or the Commission on Accreditation of Rehabilitation Facilities, and (4) the metropolitan statistical area (rural or urban status).

### Implementation strategies

The structure of the four NIATx200 implementation strategies represented clinic participation levels. Interest circles involved monthly multi-clinic teleconferences for a total of 18 direct contact hours (18 calls, each one hour in length), and allowed change teams from participating clinics to receive advice from peers and learn new skills. The learning session strategy consisted of three face-to-face multi-day sessions held approximately every six months, which were led by a core faculty team and utilized a common curriculum to offer didactic and experiential learning opportunities. The first learning session consisted of 8.5 h of content delivered over a single day, while another 13 content hours were delivered over 1.5 days during each of the second and third learning sessions, resulting in a potential for 34.5 total direct contact hours. Clinics assigned to the coaching strategy received a one-day, 4-h, site visit, as well as participated in monthly one-hour coaching calls; 22 direct contact hours were possible for the coaching strategy. On the calls, the coach and change leader, executive sponsor, and change team reviewed the impact of organizational changes to improve the study outcomes, discussed successes, and identified ideas for future change projects. The combination strategy involved the interest circle calls, coaching, and learning sessions, and consisted of a cumulative possibility of 74.5 direct contact hours. NIATx200 results indicated that clinics assigned to interventions with higher participation hours, where interest circles were the referenced intervention, showed greater improvements in wait time and admissions. As such, staff in the clinics assigned to the interventions with more opportunities to participate in the intervention and be exposed to sustainability concepts would have higher perceptions about the likelihood that changes would be sustained.

### Outcomes and measurement

The NIATx200 initiative utilized the British National Health Services Sustainability Index (BNHS-SI) to assess staff perceptions about the likelihood that a change will be sustained in the organization [23, 24]. The BNHS-SI has been utilized across multiple healthcare settings to assess staff perceptions about the sustainability of an organizational change [24, 35–45] and as a qualitative framework to qualitatively identify factors associated with the concept of sustainability [44, 46–48].

The tool (see Additional File 2 for questions) consists of 10 factors designed to assess overall staff perceptions about sustainability as well as their perceptions across three domains:

1. Process– benefits beyond helping patients, credibility of the benefits, adaptability of the improved process, and effectiveness of systems to monitor progress.
2. Staff– staff involvement and training to sustain the process, staff attitudes toward sustaining the change, senior leadership engagement, and clinical leadership engagement.
3. Organization– fit with organization’s strategic aims and culture, and infrastructure for sustainability.

The BNHS-SI utilizes an additive, multi-attribute, utility model to summarize the scores across the three domains (see Additional File 3) which are then totaled to arrive at an overall organization sustainability propensity score [24].

**Fig. 1:**
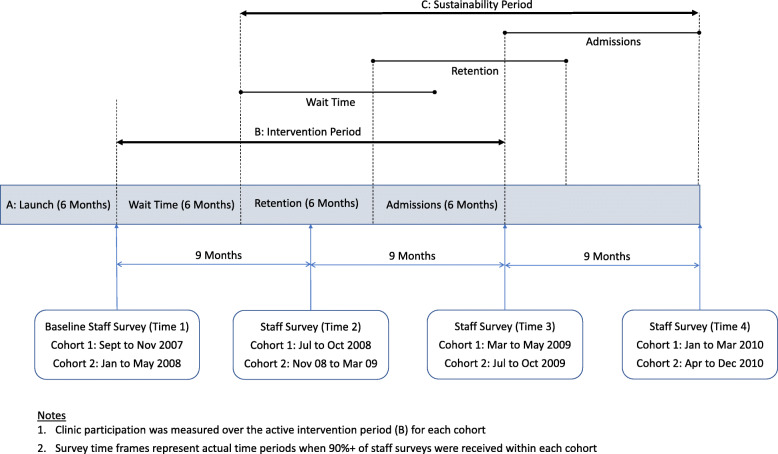
NIATx 200 Study Timeline

In the NIATx200 initiative, a staff sustainability survey [31] was developed and distributed at baseline and at every subsequent 9-month period (see Fig. 1) to prospectively assess clinic staff perceptions about sustainability capacity. The BNHS-SI measures the likelihood that a change will be sustained; therefore, it does not rely on a set sustainability definition (e.g., clinic continued to maintain the intervention after funding ended) when asking staff to complete the instrument. Instead, survey instructions stated that the BNHS-SI was “designed to gauge your organization’s propensity for sustaining changes”. As such staff were asked to “think about one specific change implemented as part of the NIATx200 project”; and then select one of four options for each of the 10 factors that best describes sustainability in their organization. Our team utilized a similar approach when assessing sustainability capacity within the Veterans Administration [38–40].

For this analysis, the cumulative extent of staff beliefs that changes implemented as part of the NIATx200 initiative would be sustained (called the Total Sustainability Score) was the primary outcome. Three secondary outcomes also were evaluated – representing scores from the process, staff, and organization domains from the BNHS-SI tool (called Process, Staff, and Organization Domain Scores, respectively).

### Data collection

Staff were invited to complete a paper survey or use a link in the invitation letter to complete the survey online. The survey also collected staff demographic information related to job function, employment status, and tenure within the organization. Two additional questions (i.e., “What is the first initial of your mother’s maiden name?” and “On what day of the month is your birthday?”) were combined with staff demographic characteristics and the clinic ID to create a unique identifier for individual staff members that allowed matching of individual survey responses to be tracked over time.

Clinic participation (direct contact hours) in the assigned implementation strategy and the number of persons from the clinic participating in the assigned implementation strategy were recorded in real time by NIATx200 research staff and coaches.

### Design and sample

The unique identifier was utilized to match individual survey responses across the four different time points (Fig. 1). As a result, all analyses are based on responses from the same staff members (*n* = 908, representing 2329 total cases) across the evaluation timeframe.

An important variable for this analysis is each clinic’s cumulative level of staff participation in QIC activities throughout the 27-month intervention interval (at baseline and approximately every nine months) (called Total Participation). Participation in each of the four study interventions was measured separately but, for the purpose and this study, was aggregated into a Total Participation metric. This variable is used to determine the influence of the number of encounters with the implementation strategy during the 27-month period. Although this factor does not represent the total number of staff who took part in each activity, and therefore reflects a clinic-level influence, it remains dependent on overall staff involvement. As such, degree of staff participation is considered appropriate and relevant, and is retained for this sample.

### Analysis

Analysis comprised both simple descriptive statistics and multivariate model building. Descriptive statistics were calculated for all variables used for this study. The type of descriptive values depended on whether the variables were continuous or categorical. For continuous variables, the mean, standard deviation (SD), and min/max are reported, while the frequencies of each category are provided for the categorical variables. Bivariate analyses were conducted on the primary outcome measure (Total Sustainability Score) and each anticipated study variable before entry into model; all variables used in the model demonstrated a significant independent association with the sustainability total.

The multivariate method was a linear mixed model repeated measures analysis that fit three separate statistical models to assess potential predictors of staff-level Total Sustainability Score, as well as on Process, Staff and Organization Domain Scores. For each model, a Repeated Covariance Type – A1(1): Heterogeneous – was used, which assumes different variances at each measurement time as well as correlations across time points that become weaker over those successive assessment times. All variables were entered into the models as fixed effects, and a maximum likelihood method was used to estimate the variable parameters. The statistical models are as follows:

1. Model I – containing only the variable representing the four time points during the NIATx200 initiative (Time),
2. Model II – containing Time, plus NIATx200-provided Implementation Strategies (i.e., learning sessions, interest circle calls, coaching sessions, or service combinations) and Job Function (i.e., administrative vs. clinical), and
3. Model III – containing the variables from Models I and II, plus organizational characteristics and the cumulative extent of participation in NIATx200-provided strategies (i.e., total number of hours).

IBM SPSSv26® was used to calculate all descriptive statistics and to estimate each model by calculating the parameter estimates for fixed effects at 95% confidence intervals. This study is reported in full accordance with the StaRI checklist [Additional File 4] [49].

## Results

### Descriptive variables

The final sample size represents responses from the same staff members (*n* = 908, representing 2329 total cases) across the evaluation timeframe. The Total Participation in the NIATx200 implementation strategies represented the only continuous independent variable used in the models and ranged from no time to 64 h (mean = 26.03, SD = 18.23). Table 1 lists the category frequencies for the remaining model variables, as well as the mean Total Sustainability Scores associated with each variable category.

**Table 1:** Frequencies of Categorical Variables and Sustainability Scores Per Category

***Variables***	***Survey Responses % (N)***	***Total Sustainability Score Mean (SE)***
Time		
Time_1_	25.1 (*n* = 585)	71.91 (1.031)
Time_2_	27.0 (*n* = 629)	75.56 (0.992)
Time_3_	26.8 (*n* = 625)	78.43 (0.965)
Time_4_	21.0 (*n* = 490)	78.66 (1.094)
Implementation Strategy*		
Learning session	24.5 (*n* = 570)	74.03 (1.053)
Interest circle	21.0 (*n* = 488)	75.55 (1.159)
Coaching	26.7 (*n* = 621)	76.86 (0.963)
Combination	27.9 (*n* = 650)	77.53 (0.949)
Job Function		
Clinicians	55.4 (*n* = 1290)	74.87 (0.782)
Administrators	44.6 (*n* = 1039)	78.16 (0.714)
Non-Profit Status		
No	13.4 (*n* = 312)	72.64 (1.60)
Yes	86.6 (*n* = 2017)	76.59 (.054)
Free-Standing Status		
No	46.5 (*n* = 1083)	74.55 (0.79)
Yes	53.5 (*n* = 1246)	77.36 (0.66)
Accreditation		
No	65.3 (*n* = 1522)	76.46 (0.63)
Yes	34.7 (*n* = 807)	75.34 (0.89)
Metropolitan Statistical Area		
Rural	21.4 (*n* = 498)	77.93 (1.09)
Urban	78.6 (*n* = 1831)	75.57 (0.58)

* Within this repeated measure sample, the percent of clinics with included staff responses represented within each implementation strategy were: Interest Circle (89.8%), Coaching (86.0%), Learning Sessions (70.4%), and Combination (89.6%)

### Repeated measures model I

#### Primary outcome

Model I (see Table 2) yielded strong overall predictive significance for Time (F = 7.270, *p* < .0001), with staff perceptions about overall sustainability capacity increasing throughout most of the study.

**Table 2:** Staff -Level Sustainability Perceptions: Linear Mixed Model Results for Model 1

	Estimate	Std. Err.	df	t	Sign.	95% CI
**Total Sustainability Score (Primary Outcome)**						
*Intercept*	78.66	1.09	445	71.92	*p* = .0001	76.51–80.81
Time_4,1_	6.74	1.50	947	4.49	p = .0001	3.79–9.69
Time_4,2_	3.10	1.48	955	2.10	*p* = .036	0.20–5.99
Time_4,3_	0.23	1.46	945	0.16	*p* > .05	−2.63 – 3.09
**Process Domain Score (Secondary Outcome)**						
*Intercept*	23.88	0.35	460	68.81	p = .0001	23.20–24.56
Time_4,1_	2.07	0.48	982	4.33	p = .0001	1.13–3.01
Time_4,2_	0.99	0.47	998	2.10	p = .036	0.60–1.91
Time_4,3_	−0.65	0.45	955	−1.45	p > .05	−1.54 – 0.23
**Staff Domain Score (Secondary Outcome)**						
*Intercept*	41.46	0.63	455	65.99	p = .0001	40.22–42.69
Time_4,1_	3.04	0.87	974	3.51	p = .0001	1.34–4.74
Time_4,2_	1.37	0.85	984	1.62	p > .05	−0.29 – 3.03
Time_4,3_	0.51	0.85	980	0.60	p > .05	−1.16 – 2.18
**Organization Domain Score (Secondary Outcome)**						
*Intercept*	13.15	0.22	463	60.47	p = .0001	12.72–13.57
Time_4,1_	1.28	0.30	987	4.27	p = .0001	0.69–1.86
Time_4,2_	0.83	0.29	999	2.82	*p* = .005	0.25–1.41
Time_4,3_	0.33	0.29	988	1.13	p > .05	−0.24 – 0.90

#### Secondary outcomes

The time effect pattern for the primary outcome was also identified for the process and organizational domains. However, Staff Domain Scores evidenced a statistically significant increase only when comparing the study endpoints.

### Repeated measures model II

#### Primary outcome

Table 3 shows that Model II did not change the overall Time effect profile identified in Model I for overall staff perceptions about sustainability capacity. However, the assigned NIATx200 strategy and staff job function were significant – participation in combined services (compared to learning sessions only) and organization administrators were associated with greater perceptions about sustainability propensity.

**Table 3:** Staff -Level Sustainability Perceptions: Linear Mixed Model Results for Model 2

	Estimate	Std. Err.	df	t	Sign.	95% CI
**Total Sustainability Score (Primary Outcome)**						
*Intercept*	82.22	1.56	984	52.63	p = .0001	79.15–85.28
Time_4,1_	7.17	1.55	867	4.62	p = .0001	4.12–10.22
Time_4,2_	3.00	1.53	877	1.960	*p* = .05	−.01–6.00
Time_4,3_	0.12	1.51	890	0.08	p > .05	−2.85 – 3.08
Comb vs. learning	3.28	1.47	1842	2.228	p = .026	−6.173461
Comb vs. circles	2.15	1.53	1848	1.407	p > .05	−0.85 – 5.14
Comb vs. coaching	0.21	1.44	1850	−0.15	p > .05	−2.62 – 3.04
Job Function (clinical)	−3.50	1.07	1850	−3.27	p = .001	−5.59 – −1.40
**Process Domain Score (Secondary Outcome)**						
*Intercept*	25.20	0.49	981	51.46	=.0001	24.23–26.16
Time_4,1_	2.18	0.49	884	4.44	p = .0001	1.22–3.14
Time_4,2_	0.92	0.48	902	1.89	p > .05	−0.03 – 1.87
Time_4,3_	− 0.58	0.46	866	1.26	p > .05	−1.49 – 0.33
Comb vs. learning	0.80	0.46	1872	1.74	p > .05	−0.10 – 1.70
Comb vs. circles	0.62	0.47	1900	1.31	p > .05	−0.31 – 1.55
Comb vs. coaching	−0.28	0.45	1888	−0.62	p > .05	−1.16 – 0.61
Job Function (clinical)	−1.72	0.33	1891	−5.18	p = .0001	−2.37 – − 1.07
**Staff Domain Score (Secondary Outcome)**						
*Intercept*	43.16	0.91	979	47.51	p = .0001	41.37–44.94
Time_4,1_	3.35	0.90	881	3.71	p = .0001	1.58–5.12
Time_4,2_	1.46	0.88	891	1.65	p > .05	−0.27 – 3.20
Time_4,3_	0.56	0.89	889	0.63	p > .05	−1.18 – 2.30
Comb vs. learning	1.70	0.86	1892	1.97	*p* = .049	0.01–3.39
Comb vs. circles	0.87	0.89	1894	0.98	p > .05	−0.87 – 2.62
Comb vs. coaching	0.15	0.84	1896	0.17	p > .05	−1.51 – 1.80
Job Function (clinical)	−1.50	0.62	1894	−2.40	*p* = .017	−2.72 – − 0.27
**Organization Domain Score (Secondary Outcome)**						
*Intercept*	13.52	0.31	1017	42.92	*p* = .0001	12.90–14.14
Time_4,1_	1.32	0.31	903	4.22	p = .0001	0.70–1.93
Time_4,2_	0.81	0.31	908	2.64	*p* = .008	0.21–1.42
Time_4,3_	0.24	0.30	912	0.80	p > .05	−0.35 – 0.84
Comb vs. learning	0.69	0.30	1921	2.34	*p* = .019	0.11–1.27
Comb vs. circles	0.36	0.31	1932	1.17	p > .05	−0.24 – 0.96
Comb vs. coaching	−0.17	0.29	1932	−0.58	p > .05	−0.73 – 0.40
Job Function (clinical)	−0.31	0.21	1932	−1.47	p > .05	−0.73 – 0.11

#### Secondary outcomes

While the overall Time effect did not differ between Model II and Model I for the Staff Domain Score, the assigned NIATx200 strategy comparing combined services to learning sessions only and staff job function were significantly associated with perceptions about staff involvement. The Time effect for the Organization Domain Score did not change from Model I to Model II, and only the assigned NIATx200 strategy was significantly associated with perceptions related to organizational capacity to support sustainability. For the Process Domain Score, the pattern of effect for time changed somewhat between Model II and Model I, and only staff job function was significant.

### Repeated measures model III

#### Primary outcome

The addition of Total Participation levels and organizational characteristics in Model III demonstrated the importance of being in a free-standing agency and having a higher participation, as measured by direct exposure hours, on overall beliefs about sustainability, but changed the statistical profiles for other model variables (see Table 4). For example, including Total Participation resulted in staff perceptions about sustainability being statistically significant only when comparing the two study endpoints. In addition, higher Total Sustainability Scores shifted to other implementation strategies (i.e.., coaching). Finally, administrators continued to report a greater sustainability propensity than clinicians.

**Table 4:** Staff -Level Sustainability Perceptions: Linear Mixed Model Results for Model 3

	Estimate	Std. Err.	df	t	Sign.	95% CI
**Total Sustainability Score (Primary Outcome)**						
*Intercept*	73.21	3.54	1819	20.71	*p* = .0001	66.28–80.15
Time_4,1_	7.08	1.55	860	4.57	*p* = .0001	4.03–10.12
Time_4,2_	2.88	1.52	865	1.89	*p* > .05	−.12–5.87
Time_4,3_	−0.04	1.50	879	0.02	*p* > .05	−2.99 – 2.92
Comb vs. learning	−0.23	1.86	1848	0.12	*p* > .05	−3.87– 3.41
Comb vs. circles	−4.79	2.76	1843	1.74	*p* > .05	−10.20 – 0.62
Comb vs. coaching	−6.74	2.50	1842	2.69	*p* = .007	−11.65 – 1.83
Job Function (clinical)	−3.44	1.07	1849	−3.22	*p* = .001	−5.54 – −1.34
Total Participation Hours	0.18	0.06	1830	3.09	*p* = .002	0.07–0.29
Non-Profit vs. For-Profit	−2.24	1.69	1848	−1.33	*p* > .05	− 5.55 – 1.07
System Program vs. Free-Standing Program	−2.23	1.09	1849	−2.06	*p* = 0.40	−4.36 – −0.10
Non-Accredited vs. Accredited	1.32	1.16	1849	1.14	*p* > .05	− 0.96 – 3.61
Rural vs. Urban	2.09	1.31	1847	1.59	*p* > .05	−0.49 – 4.67
**Process Domain Score (Secondary Outcome)**						
*Intercept*	23.64	1.10	1856	21.40	*p* = .0001	21.47–25.80
Time_4,1_	2.16	0.49	889	4.40	*p* = .0001	1.19–3.12
Time_4,2_	0.89	0.48	901	1.85	*p* > .05	−0.06 – 1.84
Time_4,3_	−0.63	0.46	868	−1.36	*p* > .05	−1.53 – 0.28
Comb vs. learning	0.06	0.58	1894	−0.10	*p* > .05	−1.19 – 1.07
Comb vs. circles	−0.78	0.86	1890	−0.90	*p* > .05	−2.47 – 0.91
Comb vs. coaching	−1.76	0.78	1883	−2.25	*p* = .025	−3.29 – 0.22
Job Function (clinical)	−1.66	0.33	1904	−4.97	*p* = .0001	−2.31 – −1.00
Total Participation Hours	0.04	0.02	1868	2.06	*p* = .039	0.002–0.07
Non-Profit vs. For-Profit	−1.11	0.53	1898	−2.11	*p* = .035	−2.14 – −0.08
System Program vs. Free-Standing Program	−0.82	0.34	1906	−2.42	*p* = 0.016	−1.48 – −0.15
Non-Accredited vs. Accredited	0.10	0.36	1901	0.28	*p* > .05	−0.61 – 0.81
Rural vs. Urban	0.50	0.41	1896	1.22	*p* > .05	−0.30 – 1.31
**Staff Domain Score (Secondary Outcome)**						
*Intercept*	37.19	2.08	1872	17.89	*p* = .0001	33.11–41.26
Time_4,1_	3.29	0.90	886	3.64	*p* = .0001	1.51–5.06
Time_4,2_	1.38	0.88	901	1.57	*p* > .05	−0.35 – 3.12
Time_4,3_	0.47	0.89	902	0.54	*p* > .05	−1.27 – 2.21
Comb vs. learning	−0.40	1.09	1901	−0.37	*p* > .05	−2.54 – 1.74.
Comb vs. circles	−3.33	1.62	1894	−2.06	*p* = .040	−6.51 – −0.15
Comb vs. coaching	−3.96	1.47	1894	−2.69	p = .007	−6.84 – −1.07
Job Function (clinical)	−1.52	0.63	1902	−2.41	*p* = .016	−2.75 – −0.28
Total Participation Hours	0.11	0.03	1882	3.10	*p* = .002	0.04–0.17
Non-Profit vs. For-Profit	−0.58	0.99	1900	−0.59	*p* > .05	−2.53 – 1.36
System Program vs. Free-Standing Program	−0.73	0.64	1902	1.14	*p* > .05	−1.98 – 0.52
Non-Accredited vs. Accredited	1.23	0.68	1901	1.81	*p* > .05	−0.10 – 2.56
Rural vs. Urban	1.14	0.77	1901	1.48	*p* > .05	−0.38 – 2.65
**Organization Domain Score (Secondary Outcome)**						
*Intercept*	12.32	0.71	1894	17.37	*p* = .0001	10.93–13.71
Time_4,1_	1.31	0.31	896	4.20	*p* = .0001	0.70–1.92
Time_4,2_	0.79	0.30	902	2.60	*p* = .009	0.19–1.39
Time_4,3_	0.21	0.30	902	0.69	*p* > .05	−0.38 – 0.80
Comb vs. learning	0.18	0.37	1930	0.49	*p* > .05	−0.55 – 0.91
Comb vs. circles	−0.64	0.55	1925	−1.16	*p* > .05	−1.73 – 0.44
Comb vs. coaching	−1.24	0.50	1924	−2.46	*p* = .014	−2.22 – −0.25
Job Function (clinical)	−0.32	0.21	1931	−1.49	*p* > .05	−0.74 – 0.10
Total Participation Hours	0.03	0.01	1912	2.22	*p* = .027	0.003–0.05
Non-Profit vs. For-Profit	−0.22	.034	1927	−0.66	*p* > .05	−0.89 – 0.44
System Program vs. Free-Standing Program	−0.66	0.22	1931	−3.03	*p* = .003	−1.08 – −0.23
Non-Accredited vs. Accredited	0.14	0.23	1929	0.58	*p* > .05	−0.32 – 0.59
Rural vs. Urban	0.60	0.26	1928	2.28	*p* = .023	0.08–1.12

#### Secondary outcomes

The findings for the Staff Domain Score were similar to the Model III results for overall sustainability capacity. Total Participation hours strongly influenced staff perceptions about the Process and Organization Domain Scores. Participation in the coaching implementation strategy, staff who were administrators, and working in a for-profit, free-standing, facility was associated with greater sustainability propensity for the process domain. For the Organizational Domain Score, the time effect increased throughout most of the study and assignment to the coaching strategy was significant, as was being involved in a free-standing facility or an agency located in a rural setting.

## Discussion

Our study explored how the extent of participation in a QIC, based on implementation strategy assignment, was associated with staff perceptions about sustainability. The study was framed in the context of two conceptual sustainability frameworks (i.e., ISF and CFSC) with clear operational definitions using a rigorous outcome measure of sustainability capacity – the BNHS-SI. To the best of our knowledge, this study was the first to track how staff perceptions about sustainability changed longitudinally. We accomplished this by using data collected over a 27-month period from a convenience sample of responses from the same provider staff members participating in a QIC (NIATx200). In addition, the study is the first to measure the number of hours of provider participation in the assigned implementation strategy of the QIC and utilize the level of participation as a predictor of how staff perceptions about sustainability change over time.

Although NIATx200 offered minimal exposure to sustainability principals, staff perceptions about their organization’s overall SC continued throughout the QIC implementation period, with the most significant improvement occurring over the first 9-month period. However, there was a clear indication of a saturation effect for perceptions of improved sustainability, with subsequent changes in overall sustainability scores eventually showing no noticeable differences as time progressed. Similar patterns occurred for the process, staff, and organizational domains. These findings support a need to continue reinforcing the importance of sustainability within organizations, especially later during the implementation process. An evaluation of long-term sustainment in the NIATx200 initiative found that between 27 to 40% of participating clinics sustained changes for one of the three study outcomes but only 12% of the clinics sustained changes for two of the three study outcomes [45]. In some instances, improvement and subsequent sustainment occurred after the end of the active NIATx200 implementation period.

It was additionally noteworthy that there were noticeable staff differences in perceptions about whether change will be sustained within organizations. Administrators and managers were much more likely to anticipate a propensity for enhanced sustainability than were clinicians within the organization. These differences are more prominent for attributes associated with the process and staffing domains of the BNHS-SI. As a result, the implementation process requires further effort to engage clinicians to convince them that effective change is beneficial and can be both attained and sustained. Such efforts could be support through sustainability specific modules (introduced early in the collaborative) followed by a re-enforcement of the sustainability concepts. In addition, the QIC could be structured to incorporate sustainability learning sessions or a coach-led sustainability site visit to re-enforce sustainability concepts or help the organization develop a sustainability plan.

In addition to staff-level factors, organizational characteristics had some notable associations with sustainability levels. Interestingly, affiliation with a free-standing Alcohol and Drug Treatment Program had the most prevalent effect, being associated with higher total sustainability, as well as with the process and organization sustainability sub-domains. For-profit facilities demonstrated higher levels of process sustainability, while rural facilities were associated with greater organizational sustainability. Perhaps not surprisingly, none of the organizational factors were statistically related to the staff sub-domain of sustainability.

Study results suggest, as outlined in the Normalization Process Theory, that (1) staff involvement (working individually and collectively) to implement the change and (2) social processes (coherence, cognitive participation, collection action and reflexive monitoring) are associated with how an innovation is embedded, integrated and sustained within the organization [25, 50, 51]. Specifically, our results demonstrated that increased exposure promotes greater belief in sustainability, which was supported both through combinations of implementation strategies and extent of participation. When added to the multivariate statistical model, an organization’s cumulative participation level in a QIC became a principal predictor of staff SC perceptions, over-riding the effects of the other factors. This finding is consistent with prior research showing that free-standing clinics participated more in NIATx200 [33]. Indeed, the pattern of effects found for the combined implementation strategies seems to be more a function of the hours of participation; when controlling for Total Participation hours, combined strategies are not as predictive of Total Sustainability as individual strategies.

Process sustainability focuses on staff beliefs in the benefits of and the credibility of the evidence for the change; how easy it is to adapt the change to the organization; and the presence of systems to monitor change. Higher levels of process sustainability in for-profit facilities may suggest that the infrastructure (e.g., training or culture) is better suited to support the constructs associated with cognitive participation (e.g., legitimation and buy-in) as well as reflexive monitoring (e.g., monitoring implementation impact) within the Normalization Process Theory [51]. Collective Action emphasizes the organizational resources needed to support change as well as the workability of the change in the organization [25, 50, 51]. In the BNHS-SI, the idea of workability may be associated with adaptability (i.e., ease of adapting the change to fit the organization) with organizational resources being associated with the infrastructure (i.e., policies and procedures and resources) to support the sustainability of change [23, 24]. Rural treatment facilities most likely do not have the resources to invest in changes that will not be sustained and therefore, take steps to establish an infrastructure needed to support and sustain change.

The results suggest that repeat exposure in different implementation strategies to sustainability concepts may help to ingrain within staff the importance of sustaining organizational change. Given these findings, it seems possible to develop and introduce specific sustainability content (e.g., how to develop and implement a sustainability plan) within the structure of a QIC as a means to assess the impact on staff SC perceptions over time and the sustainment of organizational change. The effectiveness of a QIC with a sustainability component, compared to one without, could be evaluated within a randomized control trial that assigns organizations to the implementation strategy using baseline staff perceptions about organizational readiness or sustainability capacity.

The BNHS-SI assesses the six sustainability constructs from the CFSC that consistently have been the most commonly found in at least 75% of both the sustainability approaches (retrospective versus prospective) and level of focus (organizational versus intervention) within the CFSC framework [21]. Table 5 outlines the conceptual relationships between CFSC, and ISF constructs, the prominent attributes associated with these constructs and how these attributes are measured within the BNHS-SI. For example, staff involvement and training emphasize the need for orienting and training staff to be able to deliver the initiative successfully in the CFCS and aligns with two constructs in the ISF Process domain (i.e., partnership/engagement and training/support/supervision). However, the idea measured in the BNHS is that staff have been involved from the beginning of the change and are adequately trained to sustain the improved process. Study results indicated that each hour of total participation in the NIATx QIC increased staff perceptions, as measured by the BNHS-SI, related to these six common sustainability constructs within the CFCS. Future research can attempt to directly replicate these findings by integrating the BNHS-SI as a measurement tool within the CFCS.

**Table 5:** Comparison of Conceptually Analogous Constructs from Consolidated Framework for Sustainability Constructs (CFSC), Integrated Sustainability Framework (ISF) and the British National Health Services Sustainability Index (BNHS-SI)

***CFSC Constructs***	***ISF Constructs***	***BNHS-SI Constructs***	***Attributes Assessed***
General resources	Inner Context: Organizational resources/funding	Infrastructure for sustainability	Funding, infrastructure, staff, and time
Integration with existing programs and policies	Inner Context: Climate/culture and policies Intervention Characteristics: Fit with population and context	Organizations strategic aims and cultures	Initiatives’ fit within organizational structures, programs, and policies
Demonstrating effectiveness	Intervention Characteristics: Benefits	Benefits of the change are immediately obvious and supported by evidence	Assessing the impact of the intervention over time
Monitoring progress over time	Processes: Program Evaluation	Effectiveness of the system to monitor progress	
Stakeholder participation	Processes: Partnership/Engagement and Training/Support/Supervision	Staff involvement	Staff attitudes toward sustaining the change
Training and capacity building		Training to sustain the process	

Further research should explore exactly how these organizational characteristics independently and within the Normalization Process Theory impact participation in a QIC and staff perceptions that changes will be sustained.

Evidence suggests that factors associated with sustainability include the fit of the innovation or change within the organizational culture or a culture that encourages flexibility and adaptability [52, 53], and one items in the organizational sub-process in the BNHS-SI assesses the degree that staff perceives the innovation as fitting within the organizations’ strategic aims and culture. Our study found that staff perceptions in this sub-process changed over time and that participation was associated with the improvement. Furthermore, another study identified six guiding principles associated with the sustainability of a culture change [54]. Several enabling factors associated with these principles are assessed, in part, by the BNHS-SI, such as:

- the perceived value of organizational data (Principle: Continuously assess and learn from cultural change and assessed within the Process sub-domain in the BNHS-SI);
- willingness to relinquish control (Principle: Promote staff engagement and assessed within the Staff sub-domain in the BNHS-SI); and
- perception that the change is legitimate and credible (Principle: Align vision and action and assessed within the Process sub-domain in the BNHS-SI).

The association of these factors with sustained cultural change would suggest that staff perceptions of organizational sustainability might be associated with the organizational culture that drives those perceptions. However, we did not assess either the changes in organizational culture or a relationship between culture and staff perceptions about sustainability. Future research should explore the role of organizational culture on staff perceptions about sustainability.

### Strengths and limitations

Several strengths characterize this study. First, the study design involved data collection at four points in time, allowing prospective analyses. Second, the use of a unique identifier algorithm permitted a repeated measures statistical approach. The same cases could be tracked for changes across time, allowing for a profile of the evaluation of individuals’ perceptions about sustainability. Finally, the structure of the implementation strategies within the NIATx200 initiative allowed for clinic-level participation to be tracked within the assigned implementation strategy.

Although this study had strengths, certain limitations also need to be considered. First, as mentioned previously, the data collected do not directly represent staff-level participation in QIC activities. Participation was calculated for each clinic, but still ultimately depended on staff involvement. Second, although repeated-measures analysis allowed for profiles of unique, individual, cases across time, case variation between the time phases might have existed. However, the repeated covariance model likely lessened the impact of such variation. Third, while the study comprised a 27-month intervention timeframe, consisting of baseline measurement and three 9-month intervals, there was some variation in the 9-month periods when staff surveys were completed (Fig. 1). Fourth, the sample included only responses from linked staff surveys over the four data collection time periods. Although we had staff responses from 83.6% of the participating clinics, the exclusion of certain clinics precluded controlling for successful improvement in the outcomes (wait time, retention, or admission) or exploring how success could influence staff attitudes towards sustainability. Finally, the data represented limited staff demographic information, related to employment only. Further application of the NIATx framework, which utilizes a broader variety of staff characteristics, could provide additional insights into their influences on sustainability propensity.

## Conclusion

Sustainability of organizational change represents an increasingly important focus of implementation research. Research has shown that the scientific evidence for how staff perceptions about organizational sustainability capacity, as well as what influences changes over time, represent a gap in dissemination and implementation research. These findings addressed this recognized gap in the literature. Although staff perceptions about sustainability capacity changed over time, this analysis determined that staff participation, representing the level of involvement in the assigned implementation strategy, is the most significant contributor that influenced changes in staff perception about sustainability propensity over time. The impact of participation “dose exposure” on sustainability perceptions highlight the need for dissemination and implementation strategies to re-enforce concepts associated with sustainability to improve staff perceptions about the sustainability capacity of the organization.

Since a QIC is often utilized in implementation efforts, a recognition that staff participation influences staff perceptions about sustainability can inform its design and structure and could provide a foundational step toward determining how change is sustained in substance abuse clinics. The unique perspectives from this study address recognized gaps in the literature, including when and how to incorporate sustainability concepts in a QIC to increase the likelihood that changes will be sustained. Knowing that participation in a QIC influences staff perceptions, researchers and practitioners can work to improve the structure of a QIC by developing activities or interventions (e.g., develop and introduce specific sustainability content) or modify implementation strategies (e.g., coaching for sustainability). Such efforts might promote greater staff involvement or participation in the QIC. Future research could assess the impact of these changes on the relationship between how staff perceptions of sustainability capacity change over time and whether a change is successfully sustained in the organization.

## Supplementary Information

**Additional file 1.** Sustainability Construct by Assessment Timing and Level of Focus.

**Additional file 2.** Blank British National Health Service Survey.

**Additional file 3.** British National Health Services Sustainability Index Scoring Guide.

**Additional file 4.** StaRI Checklist.

## Data Availability

Datasets generated and/or analyzed during the current study are not publicly available because the data contains potentially identifying information but are available from the corresponding author on reasonable request.

## Abbreviations

BNHS-SI: British National Health Services Sustainability Index
CFSC: Consolidated Framework for Sustainability Constructs
IRB: Institutional Review Board
ISF: Integrated Sustainability Framework
NIDA: National Institute on Drug Abuse
NIATx: Network for the Improvement of Addiction Treatment
PSAT: Program Sustainability Assessment Tool
QIC: Quality Improvement Collaborative
SC: Sustainability Capacity
