# Supplementary material for: Influence of participation in a quality improvement collaborative on staff perceptions of organizational sustainability
Source: BMC Health Serv Res. 2021 Jan 7;21:34. doi: 10.1186/s12913-020-06026-3 (PMC7791971; doi:10.1186/s12913-020-06026-3)
Supplement: Supplementary file 1 — Additional file 1. Sustainability Construct by Assessment Timing and Level of Focus. [file 12913_2020_6026_MOESM1_ESM.docx]

Appendix 1: Sustainability Construct by Assessment Timing and Level of Focus

|  | **Level of Focus** | | **Assessment Timing** | |
| --- | --- | --- | --- | --- |
| **Sustainability Construct** | Organizational | Intervention | Retrospective | Prospective |
| **Initiative design and delivery** | | | | |
| Demonstrating effectiveness | X | X | X | X |
| Monitoring programs over time | X | X | X | X |
| Training and capacity building |  | X | X | X |
| **Negotiating initiative processes** | | | | |
| Accountability of roles and responsibilities |  |  | X |  |
| Belief in the initiative | X |  | X | X |
| Defining aims and shared vision | X |  | X |  |
| **Organizational Setting** | | | | |
| Integrate w/existing programs & policies |  | X | X | X |
| Intervention adaption and receptivity |  | X |  | X |
| Organizational Readiness & Capacity | X |  |  |  |
| Organizational values & culture | X | X |  |  |
| **People Involved** | | | | |
| Community participation | X |  |  |  |
| Leadership and champions | X | X | X | X |
| Relationships and collaboration and networks |  |  |  | X |
| Stakeholder participation | X | X |  | X |
| **Resources** | | | | |
| Funding |  | X | X |  |
| Resources general | X | X | X | X |

Adapted from Lennox and colleagues.
