# Supplementary material for: Influence of participation in a quality improvement collaborative on staff perceptions of organizational sustainability
Source: BMC Health Serv Res. 2021 Jan 7;21:34. doi: 10.1186/s12913-020-06026-3 (PMC7791971; doi:10.1186/s12913-020-06026-3)
Supplement: Supplementary file 3 — Additional file 3. British National Health Services Sustainability Index Scoring Guide. [file 12913_2020_6026_MOESM3_ESM.pdf]

NHS Sustain**ability** Model

Authors

Lynne Maher[1]   David Gustafson[2]   Alyson Evans[2]

Directions

Read through the model.
Select the level of each factor that best describes your situation.
Circle or mark your score.
Add the scores from each factor level that you selected and enter into the assessment panel at the bottom.

Scores

Preliminary evidence suggests; a score of 55 or higher offers reason for optimism while a score of 45 or lower suggests that you need to take some action to increase the likelihood that your improvement initiative will sustain.

Look initially at the factors that you scored with lower marks. You will find some useful information in the corresponding section of this guide which will help you to devise an action plan for improvement.

You will find it helpful to continue to use the model over time and we suggest reviews at periods of three to six months.

We are continuing to assess the use and impact of the sustainability model. We would be pleased to receive any thoughts or comments that you have for improvement.

1 - Modernisation Agency of the British National Health Service, 4th Floor, St Johns House. East Street, Leicester LE1 6NB
2 - University of Wisconsin, Rm 1119 WARF Building, 610 Walnut Street, University of Wisconsin Madison 53705

Process

Choose the **Factor Level** that comes closest to your situation and circle the **Score** to the left of it

Factor	Score	Factor Level
**Benefits beyond helping patients**	8.7	The change improves efficiency and makes jobs easier
	4.7	The change improves efficiency but does not make jobs easier
	4.0	The change does not improve efficiency but does make jobs easier
	0.0	The change neither improves efficiency nor makes jobs easier
**Credibility of the benefits**	9.1	Benefits of the change are immediately obvious supported by evidence and believed by stakeholders
	6.3	Benefits of the change are not immediately obvious even though they are supported by evidence and believed by stakeholders
	3.1	Benefits of the change are not immediately obvious even though they are supported by evidence. They are not believed by stakeholders
	0.0	Benefits of the change are neither immediately obvious supported by evidence nor believed by stakeholders
**Adaptability of improved process**	7.0	The process can be adapted to other organisational changes and there is a system for continually improving the process
	3.4	The process can be adapted to other organisational changes but there is no system for continually improving the process
	2.4	The process is not able to adapt to other organisational changes but there is a system for continually improving the process
	0.0	The process is not able to adapt to other organisational changes and there is no system for continually improving the process
**Effectiveness of the system to monitor progress**	6.7	There is a system in place to identify evidence of progress, monitor progress, act on it and communicate results.
	3.3	There is a system in place to identify evidence of progress and act on it, but the results are not communicated
	2.4	There is a system in place to identify evidence and monitor progress. The results are communicated but no one acts on them
	0.0	There is no system in place to identify evidence of progress or to monitor progress nor act or communicate it

Process Total Score						
	1st Assessment	Date	2nd Assessment	Date	3rd Assessment	Date

Staff

Choose the **Factor Level** that comes closest to your situation and circle the **Score** to the left of it

Factor	Score	Factor Level
**Staff involvement and training to sustain the process**	11.5	Staff have been involved from the beginning of the change and adequately trained to sustain the improved process
	4.9	Staff have been involved from the beginning of the change but not adequately trained to sustain the improved process
	6.3	Staff have not been involved from the beginning of the change but they have been adequately trained to sustain the improved process
	0.0	Staff have neither been involved from the beginning nor adequately trained to sustain the improved process
**Staff attitudes toward sustaining the change**	11.0	Staff feel empowered as part of the change process and believe the improvement will be sustained
	5.1	Staff feel empowered as part of the change process but don't believe the improvement will be sustained
	5.1	Staff don't feel empowered by the change process but believe the improvement will be sustained
	0.0	Staff don't feel empowered by the change process or believe the improvement will be sustained
**Senior leadership engagement**	15.0	Organisational leaders take responsibility for efforts to sustain the change process and staff generally share information with and actively seek advice from the leader
	6.2	Organisational leaders don't take responsibility for efforts to sustain the change process but staff generally share information with and seek advice from the leader
	5.7	Organisational leaders take responsibility for efforts to sustain the change process but staff typically don't share information with or seek advice from the leader
	0.0	Organisational leaders don't take responsibility for efforts to sustain the change process and staff typically do not share information with or seek advice from the leader
**Clinical leadership engagement**	15.0	Clinical leaders take responsibility for efforts to sustain the change process and staff generally share information with and actively seek advice from the leader
	6.7	Clinical leaders don't take responsibility for efforts to sustain the change process but staff generally share information with and actively seek advice from the leader
	5.5	Clinical leaders take responsibility for the efforts to sustain the change process but staff typically do not share information with or actively seek advice from the leader
	0.0	Clinical leaders don't take responsibility for efforts to sustain the change process and staff typically do not share information with or actively seek advice from the leader

Staff Total Score						
	1st Assessment	Date	2nd Assessment	Date	3rd Assessment	Date

Organisation

Choose the **Factor Level** that comes closest to your situation and circle the **Score** to the left of it

Factor	Score	Factor Level
**Fit with the organisation's strategic aims and culture**	7.2	There is a history of successful sustainability improvement goals are consistent with the organisation's strategic aims
	3.3	There is a history of successful sustainability but the improvement and organisation is strategic aims are inconsistent
	3.5	There is no history of successful sustainability but the improvement goals are consistent with the organisation's strategic aims
	0.0	There is no history of successful sustainability and the improvement goals are inconsistent with the organisation's strategic aims
**Infrastructure for sustainability**	9.7	Staff, facilities and equipment, job descriptions, policies, procedures and communication systems are appropriate for sustaining the improved process
	4.4	There is an appropriate level of staff, facilities and equipment, but inadequate job descriptions, policies, procedures and communication systems for sustaining the change
	3.3	The levels of staff, facilities and equipment to sustain the change are not appropriate although job descriptions, policies, procedures and communication systems are adequate
	0.0	The staff, facilities and equipment, job descriptions, policies and procedures and communication systems are all not appropriate for sustaining the change

**Organisation Total Score**	1st	2nd	3rd
+			
**Staff Total Score**	1st	2nd	3rd
+			
**Process Total Score**	1st	2nd	3rd
=			
**Sustainability Total Score**			
	1st Assessment · Date	2nd Assessment · Date	3rd Assessment · Date

How to calculate your score

Add the **Process**, **Staff** and **Organisation** scores together and place in the **Sustainability Total Score** box.

The closer your score is to 100, the better chance of successful sustainability.

55 or higher offers reason for optimism

45 or lower suggests reason for concern
